# Randomized investigation of the bioavailability of fluoride in saliva after administration of sodium fluoride, amine fluoride and fluoride containing bioactive glass dentifrices

**DOI:** 10.1186/s12903-019-0805-6

**Published:** 2019-06-18

**Authors:** Ella A. Naumova, Moritz Staiger, Ouafaa Kouji, Jakov Modric, Thessa Pierchalla, Maya Rybka, Robert G. Hill, Wolfgang H. Arnold

**Affiliations:** 1 Department of Biological and Material Sciences in Dentistry, Faculty of Health, Witten/Herdecke University, Alfred-Herrhausen-Strasse 44, 58455 Witten, Germany; 2 Institute of Dentistry, Barts and The London School of Medicine and Dentistry, Queen Mary University of London, London, E1 4NS UK

## Abstract

**Objectives:**

Bioactive glasses which degrade in aqueous solutions may release bioactive ions such as fluoride (F^-^) and support fluoride bioavailability in saliva. We investigated how these effects would be apparent in an in vivo experimental trial after toothbrushing in comparison with sodium fluoride and amine fluoride.

**Material and methods:**

In this single-center, randomized, parallel in vivo trial with a three strata block design, where healthy subjects were randomly assigned into three groups. Each group brushed their teeth either with fluoridated bioactive glass containing dentifrice, with a sodium fluoride (NaF) containing dentifrice or with amine fluoride (AmF) containing toothpaste. Saliva was collected time intervals before, immediately after, 30, 60 and 120 min after toothbrushing. Fluoride concentration was determined in supernatant saliva and salivary sediment using a fluoride ion selective electrode. The data were evaluated statistically using non-parametric tests.

**Results:**

The increase of bioactive fluoride in supernatant saliva was higher after application of NaF or AmF compared to fluoridated bioactive glass. In salivary sediment bioavailability of fluoride lasted longer after application of fluoridated bioactive glass.

**Conclusions:**

Toothbrushing with the fluoride containing bioactive glass dentifrices had positive effects on the fluoride bioavailability within two hours. Fluoride containing bioactive glass represent a new area for investigation in caries prophylaxis. The bioactive potential impact on the tooth remineralization should be examined further.

**Trial registration:**

DRKS00016038.

## Introduction

Fluoride is a key factor in both dental restorative and oral healthcare products [1]. Numerous studies demonstrated the effectiveness of fluoride in caries prevention and it is now acknowledged that fluorides are the most effective agents in caries prevention [1–4]. Fluorides enhance enamel surface remineralization, reducing its susceptibility to demineralization [5–9], increase the resistance of the apatite structure to acid attack and have antibacterial properties [10]. Because dentifrices are widely used in oral hygiene and caries prevention fluoride compounds are added to the majority of them. Various fluoride compounds are used in the different dentifrices of which the most common in Europe are sodium fluoride (NaF) and amine fluoride (AmF). The caries preventive effect of fluoride is dependent on the fluoride bioavailability [11].

However, the bioavailability of fluoride after tooth brushing is limited to a relatively short time interval. It has been shown, that the salivary fluoride level is back to baseline after tooth brushing with conventional fluoride containing dentifrices after 120 min [12–15]. On the other hand, high fluoride doses are toxic and may result in enamel disruption such as fluorosis [16–18]. It has also been shown, that low doses of fluoride also enhance enamel remineralization [9]. In the light of increasing awareness in the population of the uptake of substances which may have an impact on general and oral health it is reasonable to reduce their uptake. Therefore, attempts to increase the bioavailability of fluoride in the oral cavity for a longer time period and to reduce the fluoride dose at the same time have been undertaken. Materials releasing calcium and fluoride ions for dentin and enamel remineralization have been the topics of intensive research [19]. A novel development is fluoride-containing bioactive glass (F-bioactive glass) with a relative low fluoride content compared to the conventional fluoride containing dentifrices [20–22]. F-bioactive glasses are amorphous silicate glasses, which degrade in aqueous solutions [10, 23]. F^−,^bioactive glass acts in aqueous solutions as a single source of both calcium, phosphate and fluoride ions [19]. During selective dissolution they may release bioactive ions such as fluoride, strontium, or calcium [20, 24]. After hydrolysis, selective dissolution and the ion exchange of F^−^bioactive glass in an aqueous environment apatite can precipitate on biological surfaces and elicit an interfacial biological response, such as bioactive fixation, resulting in inhibiting further dissolution, and decrease complete resorption of the material [10]. Bioactive glasses are biocompatible and effective for bone regeneration, bone engineering [25, 26] and for the application in the oral cavity [27].

In medicine bioactive glasses have been developed for the treatment of osteoporosis by substitution of calcium with strontium [28] and for the application in orthopedic surgery [25]. Strontium substituted bioactive glass increases osteoblast proliferation and alkaline phosphatase activity [28]. Fluoride-containing bioactive glasses also impact osteoblast behavior and activity, enhance and control of their proliferation, differentiation and mineralization [10]. Bioactive glasses release bioactive ions slowly which results in a prolonged biological effect [25]. Because of the ability to release fluoride locally the fluoride-containing bioactive glasses have already been used in dentistry for various clinical applications e. g. for the treatment of dentin hypersensitivity [10, 29]} and for regeneration of osseous defects of the alveolar ridge [10, 22, 25, 30]. It has also been shown in in-vitro experiments, that F-Bioactive glass induces fluor-hydroxyapatite formation on enamel surfaces [21, 22, 31, 32]. Another preliminary study demonstrated, that fluoride-containing bioactive glass enhances enamel remineralization [21]. Bioactive glasses can bond to hard and soft tissues [10]. However, to our knowledge there are no comparative clinical studies about the bioavailability of fluoride in oral cavity after toothbrushing with a F-bioactive glass containing dentifrice. It was therefore the aim of this in-vivo study to investigate the dynamics of fluoride bioavailability in salivary compartments (supernatant and sediment) up to two hours after standardized toothbrushing with F-bioactive glass dentifrice and compare it with the fluoride bioavailability in salivary compartments after application of dentifrices containing NaF and AmF. The null hypothesis of this study assumed that there are no differences in the fluoride bioavailability between the different dentifrices in supernatant saliva and salivary sediment.

## Material and methods

Prior to the investigation the study has been approved by the ethical committee (Nr. 170/2016) of Witten/Herdecke University. This study has been registered in the German Clinical Trials Register (# DRKS00016038). Registration was done after the study has been conducted and the results suggested a publication and further continuation of this research. The authors confirm that all ongoing and related trials for this drug/intervention are registered. All experiments were performed in accordance with relevant guidelines, and informed written consent was obtained from all participants.

### Test subjects

**Fig. 1:**
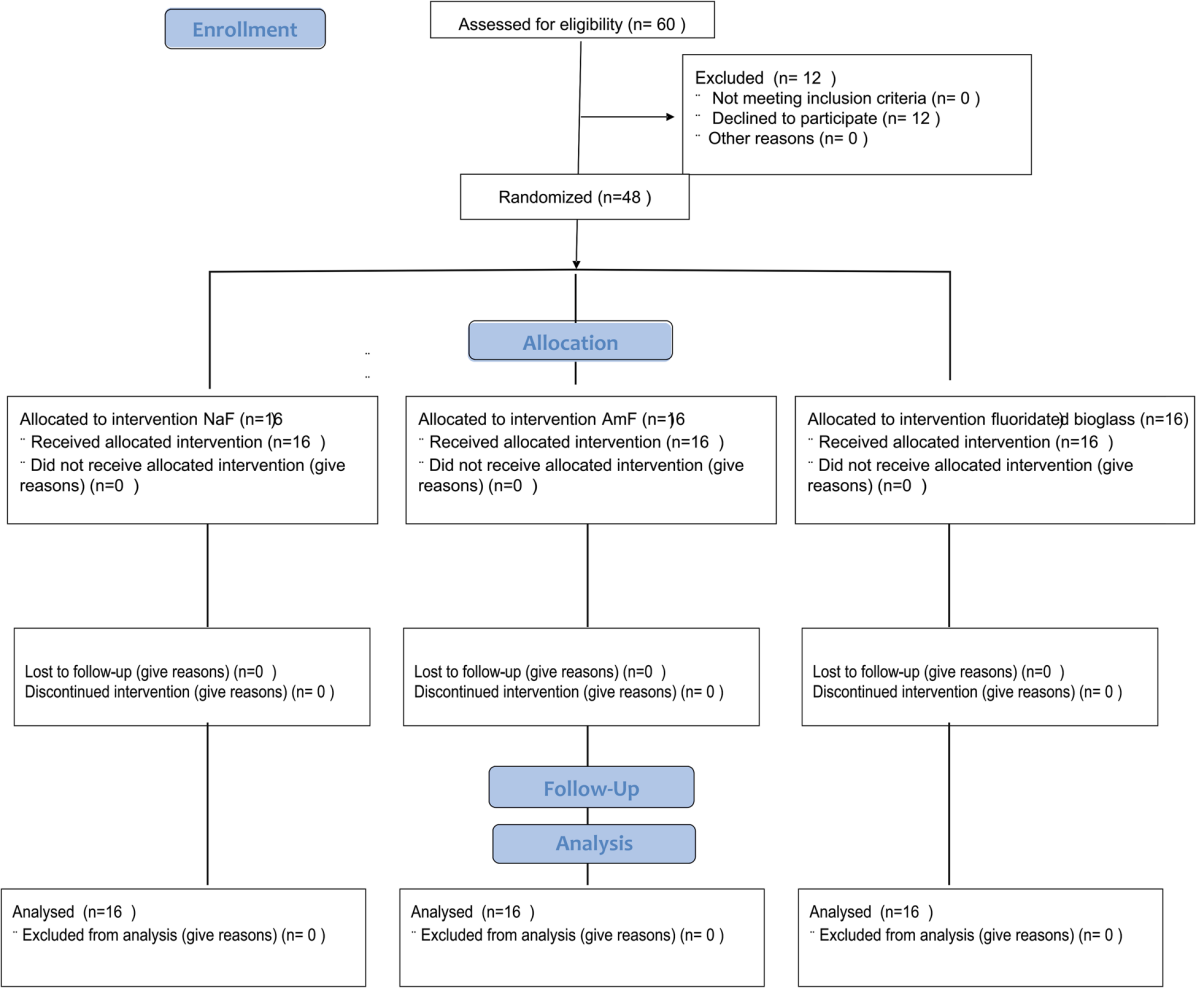
CONSORT flow chard depicting the three study arms

Sixty subjects were asked to participate in this study. Twelve refused to participate. Forty eight test subjects aged between 20 and 28 years finally participated in this parallel study (Fig. 1). All participants received written instructions and a schedule. Participants were further asked to avoid fluoride-rich food products such as tea, fish and specified mineral water during the period but had no restriction concerning drinking water. All test subjects were residents in the area with ≈ 0.2 ppm fluoride in the drinking water and normally used fluoride containing dentifrices twice daily. They were randomly and evenly distributed into three groups of eight male and eight female subjects. All subjects received verbal and written information about the investigation, as well as written instructions regarding the schedule of the study and proper tooth brushing methods. The inclusion criteria were satisfactory oral and general health. The exclusion criterion was the presence of active caries or periodontal disease and systemic disease.

### Materials

Three commercially available dentifrices with three different fluoride formulations, F-bioactive glass (530 ppm) containing dentifrice (BioMin F®; BIOMIN, London, UK), a NaF (1450 ppm) containing dentifrice (Eurodont, MAXIM Markenprodukte, Pulheim, Germany) and an AmF (1450 ppm) containing toothpaste (Elmex, CP Gaba, Hamburg, Germany) were used for toothbrushing in the present study. The ingredients of the dentifrices are listed in Table 1.

**Table 1:** Ingedients of the detifrices

Name	Active fluoride component	Other ingredients
BioMin F	Bioactive glass	Glycerin;
		Silica;
		PEG 400;
		Fluoro Calcium Phpspho Slicate;
		Sodium Lauryl Sulphate;
		Titanium Dioxide;
		Aroma;
		Carbomer;
		Potassium Acesulfame
eurodont	NaF	Aqua;
		Hydrated silica;
		Sorbitol;
		Propylene Glycol;
		NaF;
		Potassium Nitrate;
		Sodium Cq4–16 Olefin;
		Sulfonate;
		Aroma;
		Cellulose gum;
		Sodium Saccarin
Elmex	AmF	Aqua;
		Hydrated silica;
		Sorbitol;
		Hydroxyaethylcellulose;
		Olafluor;
		Aroma;
		Limonene;
		Cl 77,891;
		Sodium;
		Saccarin;
		Hydrochloric acid

### Study design and sample collection

The study was carried out as a parallel study and done at 2 .p.m. for each experiment. Prior to the experiments salivary flow rate was determined and only normal secretors (0.25–1.0 g/min) were included. For standardization of tooth brushing all test subjects brushed their teeth with the same technique (Bass’ method of tooth brushing) [33]. Saliva was collected before (T0), immediately after (T1), 30 (T2), and 120 (T3) minutes after tooth brushing by spitting into a plastic tube. Mouth rinsing was conducted immediately after collection of sample T1 with 10 ml tap water within 10 s. After a washout period of two weeks the groups repeated the brushing cycle. All experiments were repeated three times. After collection saliva was centrifuged (B Centrifuge, Beckman Coulter GmbH, Krefeld, Germany) at a speed of 3024 x g for 10 min. in micro-centrifuge tubes to separate the cell-free supernatant saliva and the cell containing sediment. Supernatant saliva and salivary sediment were separated and frozen at − 80^0^ until fluoride determination.

### Fluoride measurement

One ml supernatant saliva was taken and mixed with 1 ml of a TISAB II buffer solution (Thermo Electron, Beverly, MA, USA). For equal fluoride ion distribution during the measurement a magnetic stick stirrer (size 2 mm × 5 mm) was used. The sediment was removed from the centrifuge tube, dispersed in 250 ml TISAB II buffer solution (Vortex-Genie 2 Scientific Industries, New York, USA) in a new tube and weighted again. The fluoride concentration was determined using a fluoride ion selective electrode (96–09 Orion, Thermo Electron, Beverly, MA, USA). The protocol of fluoride determination followed exactly as described by [15].

### Statistics

A power calculation, based on the data of a previous study [34], was performed with a power of 0.8 and α = 0.05 (mean 1 = 0.04; STD 1 = 0.084; mean 2 = 0.72; STD 2 = 0.88). The power analysis revealed a minimum sample size of 14 subjects. As program for the power analysis Axum 7 (Mathsoft, Cambridge, Massachusetts, USA) was used.

From the repeated measurements of every subject the mean value was calculated for each measurement and used for statistical evaluation. The Shapiro-Wilk and Kolmogorov-Smirnov test have been used for testing the normality of the data. As these tests were negative the results were evaluated statistically with the non-parametric Mann-Whitney U test for independent variables and the Wilcoxon sign test for related variables. Descriptive statistical data were presented as boxplot graphics and tables. As statistic program served Graphpad Prism Ver. 7.0 (Graphpad, La Jolla, Ca, USA).

## Results

### Supernatant saliva

**Fig. 2:**
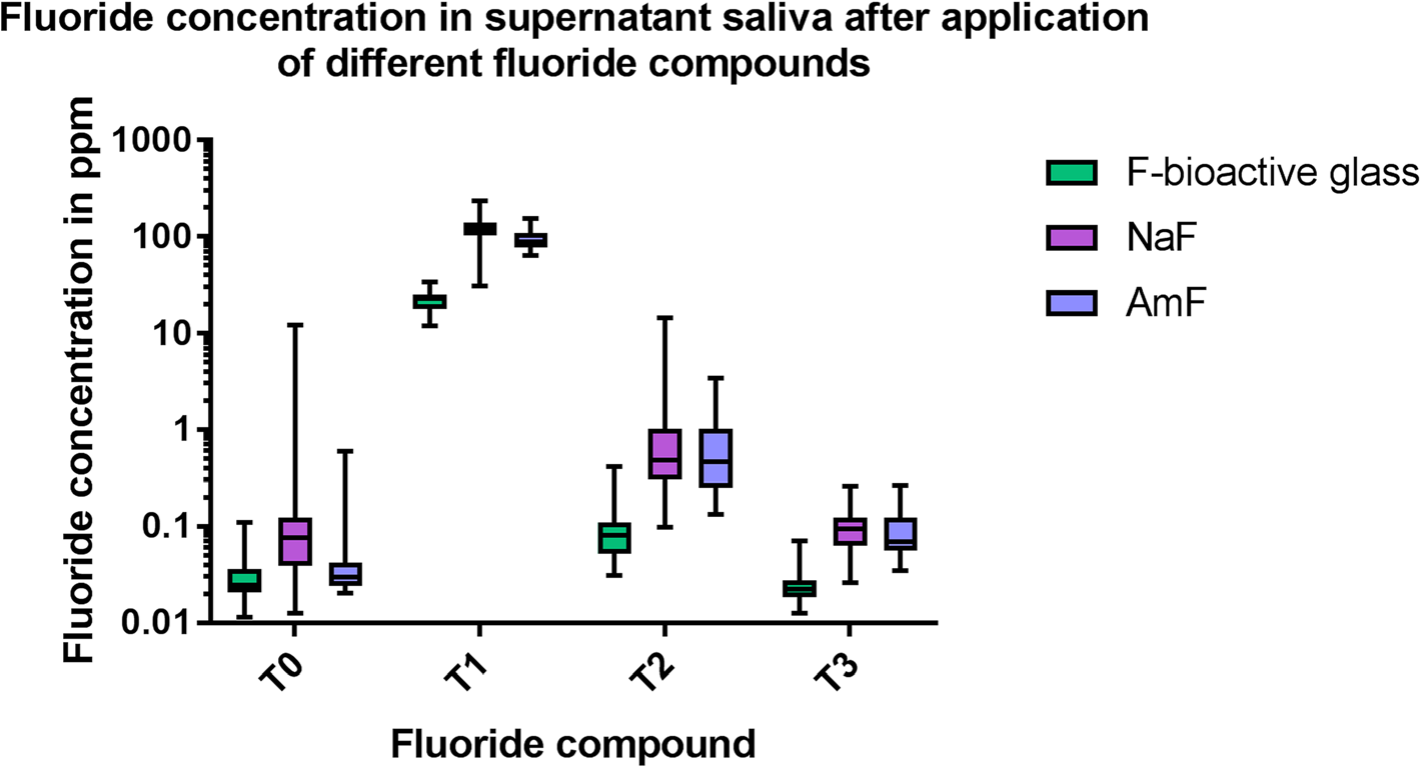
Boxplot graphics of the data distribution of the fluoride content in supernatant saliva after application of different fluoride compounds at different collection times (y axis log scale)

Comparison of the fluoride content in supernatant saliva at the different collection times revealed statistically significant differences between T0 and T1 for all fluoride compounds. Fluoride content was still significantly higher compared to T0 at T2 and T3 (Table 2 and Fig. 2). After NaF application the fluoride content in the supernatant saliva reached the baseline level after 120 min and in AmF after 30 min (Table 2 and Fig. 2).

**Table 2:** Fluoride content at different time points in supernatant saliva

	T0 - T1	T0 - T2	T0 - T3
F-bioactive glass	*p* < 0.001	p < 0.001	*p* < 0.001
NaF	p < 0.001	p < 0.001	*p* = 0.129
AmF	*p* < 0.001	*p* = 0.687	*p* = 1.000

Between the different fluoride compounds statistically significantly differences were found between F^−^ bioactive glass and NaF respectively AmF at T1, between F bioactive glass and NaF at T2, between F^−^ bioavtive glass and NaF respectively AmF at T3. All results are summarized in Table 3. All descriptive data of the distribution of the measured values of supernatant saliva are summarized in Table 4.

**Table 3:** Differences between fluoride content in different fluoride compounds at different collection times in supernatant saliva

T0		
	F-bioactive glass	AmF
NaF	p < 0.001	p < 0.001
AmF	*p* = 0.217	
T1		
	F-bioactive glass	AmF
NaF	p < 0.001	*p* = 0.024
AmF	p < 0.001	
T2		
	F-bioactive glass	AmF
NaF	p < 0.001	p < 0.001
AmF	*p* = 0.246	
T3		
	F-bioactive glass	AmF
NaF	p < 0.001	p < 0.001
AmF	*p* = 0.036

**Table 4:** Descriptive data of the measured values of fluoride content in supernatant saliva in ppm

	Median	Minimum	Maximum
T0			
F-bioactive glass	0.024	0.01	0.11
NaF	0.07	0.01	12.27
AmF	0.04	0.01	2.51
T1			
F-bioactive glass	23.05	12.00	33.73
NaF	122.83	30.70	234.67
AmF	109.00	66.33	168.33
T2			
F-bioactive glass	0.08	0.03	0.41
NaF	0.48	0.10	14.50
AmF	0.34	0.09	6.32
T3			
F-bioactive glass	0.02	0.01	0.07
NaF	0.09	0.03	0.26
AmF	0.07	0.02	0.76

### Salivary sediment

**Fig. 3:**
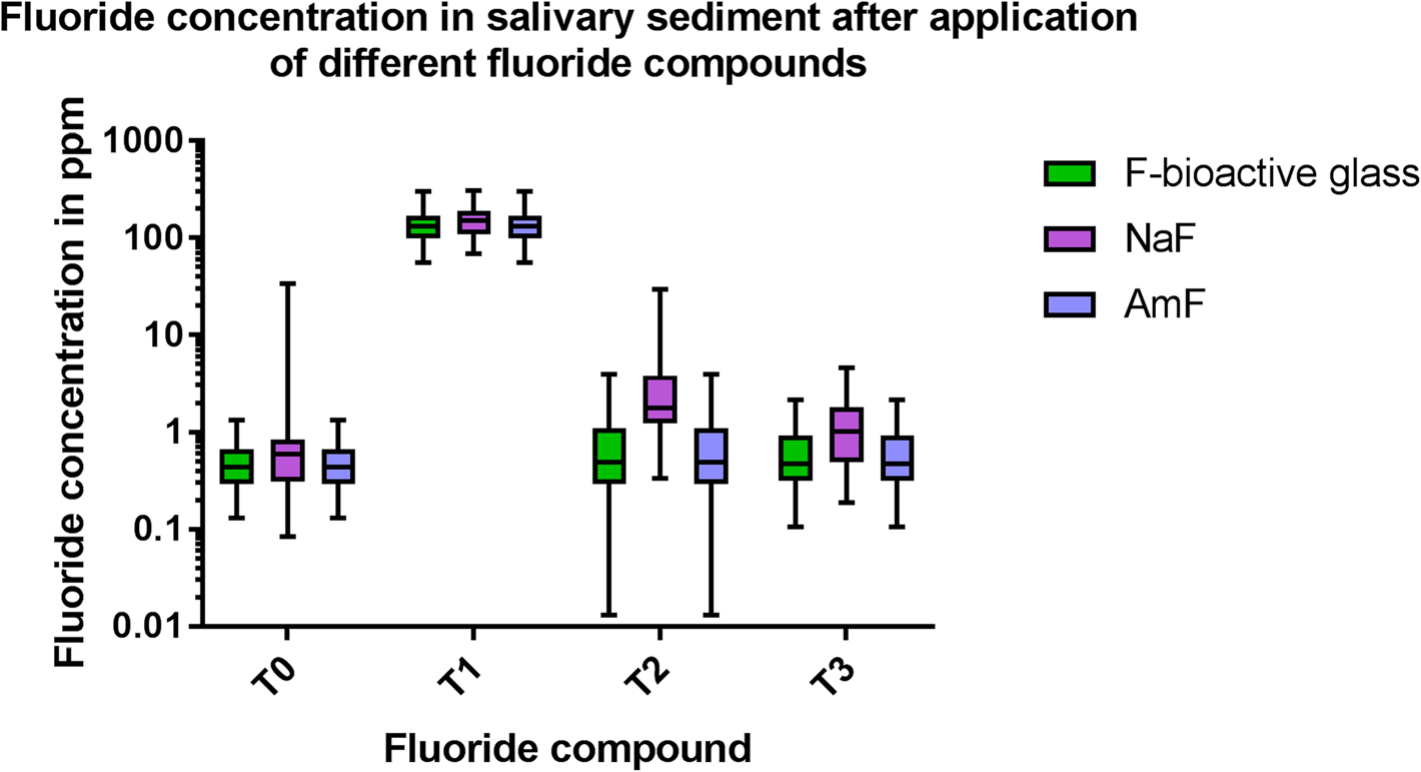
Boxplot graphics of the data distribution of the fluoride content in salivary sediment after application of different fluoride compounds at different collection times (y axis log scale)

Comparison of the fluoride content in salivary sediment at the different collection times revealed statistically significant differences between T0 and T1 for all fluoride compounds. Fluoride content was still significantly higher compared to T0 at T2 in NaF and AmF. but not for F-bioactive glass. Between T0 and T3 a significant difference was found only for AmF (Table 5 and Fig. 3).

**Table 5:** Fluoride content at different time points in salivary sediment

	T0 - T1	T0 - T2	T0 - T3
F-bioactive glass	p < 0.001	*p* = 0.303	p = 0.246
NaF	*p* < 0.001	p < 0.001	*p* = 0.012
AmF	*p* < 0.001	p < 0.001	p < 0.001

In salivary sediment the fluoride content was significantly lower after AmF application than after F-bioactive glass or NaF application (Table 6). At one collection point T2 a significant difference between all fluorides compounds was observed. The NaF content was higher than with the other compounds (Table 6.). At collection point T3 no difference in the fluoride content was observed between F-bioactive glass and AmF (Table 5. and Fig. 3). All descriptive data of the distribution of the measured values of salivary sediment are summarized in Table 7.

**Table 6:** Differences between fluoride content in different fluoride compounds at different collection times in sliavary sediment

T0		
	F-bioactive glass	AmF
NaF	*p* = 0.247	p < 0.001
AmF	p < 0.001	
T1		
	F-bioactive glass	AmF
NaF	*p* = 0.265	p < 0.001
AmF	p < 0.001	
T2		
	F-bioactive glass	AmF
NaF	p < 0.001	p < 0.001
AmF	p < 0.001	
T3		
	F-bioactive glass	AmF
NaF	p < 0.001	p < 0.001
AmF	*p* = 0.002

**Table 7:** Descriptive data of the measured values of fluoride content in salivary sediment in ppm

	Median	Minimum	Maximum
T0			
F-bioactive glass	0.44	0.13	1.34
NaF	0.59	0.08	33.93
AmF	0.03	0.02	1.90
T1			
F-bioactive glass	131.84	33.82	300.91
NaF	149.76	68.16	307.59
AmF	19.07	2.26	74.83
T2			
F-bioactive glass	0.81	0.01	3.91
NaF	1.77	0.34	29.77
AmF	0.72	0.3	3.91
T3			
F-bioactive glass	0.47	0.11	2.18
NaF	1.02	0.19	4.61
AmF	0.22	0.04	2.04

## Discussion

The most important unmet need in caries disease prophylaxes is the achievement of apatite demineralization-remineralization balance. The protection of apatite from destruction can be achieved by the application of the protective substances that can slow or prevent tooth demineralization. Force the remineralization or decrease the development of the oral biofilm with the pathogenic microorganisms. Which produce acid for the HAP destruction. Fluoride has all three of these characteristics: stabilizing the demineralization-remineralization balance [35, 36]. decreasing the development of the plaque [37] and has antibacterial activity against streptococci mutans [38]. which are accumulated in the oral biofilm [12].

Fluoride occurs in the oral cavity in two forms: active (free fluoride ions) and inactive (bound or complexed fluoride). depending on the pH. ionic strength and protein concentration; these two forms are often easily interchangeable by simply changing the pH [39]. The active form of fluoride determines its bioavailability.

After application of fluoride containing dentifrices the fluoride bioavailability in saliva increases dramatically for a short time interval and then declines reaching the baseline level after about 120 min [13, 15, 34, 40–42]. Several studies demonstrated that the content of fluoride in supernatant saliva is much lower than in the salivary sediment [14, 15]. NaF dissolves in saliva quickly and has a rapid clearance because it is swallowed with saliva. The amino group of AmF is supposed to adhere to organic surfaces and remain longer in the oral cavity. Not much is known about the bioactive potential of the F-bioactive glass in the oral cavity [10]. So far there is no in-vivo *study* about the distribution and retention of fluoride in supernatant saliva and salivary sediment for F-bioactive glass containing dentifrice.

The primary outcome in the present study was the adjusted fluoride bioavailability dynamic in supernatant saliva and salivary sediment during two hours after standardised toothbrushing. With the fluoride-containing bioactive glasses dentifrice compared to the dentifrice with the other fluoride formulations followed by a one-week washout period.

The results of the present study showed. That there is a difference between the fluoride bioavailability in supernatant saliva and salivary sediment for all three dentifrices with the different fluoride compounds. Therefore the null hypothesis has been rejected. In supernatant saliva the fluoride bioavailability after brushing with the NaF containing dentifrice was back to baseline after 120 min whereas after AmF containing toothpaste application it was back to base line as soon as 30 min after application. Fluoride bioavailability in the supernatant saliva after F-bioactive glass dentifrice application remained significantly higher than the baseline until 120 min after application. Therefore. the original assumption. That F-bioactive glass may release fluoride for a prolonged time interval than two hours after toothbrushing might be accepted. For salivary sediment completely. Different results were obtained. Like supernatant saliva in salivary sediment the fluoride bioavailability was back to the baseline level after 120 min after application of a NaF containing dentifrice. However. after application of an AmF containing dentifrice the fluoride bioavailability remained significantly higher compared to the baseline until 120 min after application. After application of a F-bioactive glass dentifrice the fluoride bioavailability was back to baseline as soon as 30 min after application. It may be speculated that the fact that the fluoride is bound the bioactive glass and not to the proteins of the sediment is the reason for the quick clearance from salivary sediment. With respect to NaF and AmF the results confirm previous studies about the clearance of fluoride in whole saliva [43–46]. Only few studies exist about the fluoride bioavailability after application of NaF or AmF in salivary sediment [14, 15]. It has been discussed whether salivary sediment acts as fluoride reservoir releasing fluoride slowly for a prolonged time. Fluoride bioavailability varies also in the different oral niches where different surfaces influence the fluoride bioavailability [12, 47, 48]. Other studies investigated the fluoride bioavailability in dental plaque and reported elevated fluoride concentrations after toothbrushing [44, 46].

However, in contrast to both other applied fluoride compounds the fluoride bioavailability in salivary sediment could not be proven after application of F-bioactive glass. Therefore, it might be assumed. That the mechanisms for liberating bioavailable fluoride for NaF. AmF and fluoride-containing bioactive glass from salivary sediment might be completely different. Whether this has an influence on the remineralization of the enamel surface has to be elucidated in further experimental and clinical studies.

## Conclusions

Within the limits of this investigation it may be concluded that administration of F-bioactive glass with 530 ppm F^−^ results in a similar fluoride bioavailability in supernatant saliva as administration of NaF or AmF with 1450 ppm F^−^.

## Data Availability

The datasets used and/or analysed during the current study are available from the corresponding author on reasonable request.

## Abbreviations

AmF: Amine fluoride
F^−^: Fluoride
NaF: Sodium fluoride
T0: Baseline
T1: Immeditedly after fluoride application
T2: 30 Minutes after fluoride application
T3: 120 Minutes after fluoride application
